# Probing the Sophisticated Synergistic Allosteric Regulation of Aromatic Amino Acid Biosynthesis in *Mycobacterium tuberculosis* Using ᴅ-Amino Acids

**DOI:** 10.1371/journal.pone.0152723

**Published:** 2016-04-29

**Authors:** Sebastian Reichau, Nicola J. Blackmore, Wanting Jiao, Emily J. Parker

**Affiliations:** Biomolecular Interaction Centre and Department of Chemistry, University of Canterbury, Christchurch, New Zealand; University of Queensland, AUSTRALIA

## Abstract

Chirality plays a major role in recognition and interaction of biologically important molecules. The enzyme 3-deoxy-d-*arabino*-heptulosonate 7-phosphate synthase (DAH7PS) is the first enzyme of the shikimate pathway, which is responsible for the synthesis of aromatic amino acids in bacteria and plants, and a potential target for the development of antibiotics and herbicides. DAH7PS from *Mycobacterium tuberculosis* (*Mtu*DAH7PS) displays an unprecedented complexity of allosteric regulation, with three interdependent allosteric binding sites and a ternary allosteric response to combinations of the aromatic amino acids l-Trp, l-Phe and l-Tyr. In order to further investigate the intricacies of this system and identify key residues in the allosteric network of *Mtu*DAH7PS, we studied the interaction of *Mtu*DAH7PS with aromatic amino acids that bear the non-natural d-configuration, and showed that the d-amino acids do not elicit an allosteric response. We investigated the binding mode of d-amino acids using X-ray crystallography, site directed mutagenesis and isothermal titration calorimetry. Key differences in the binding mode were identified: in the Phe site, a hydrogen bond between the amino group of the allosteric ligands to the side chain of Asn175 is not established due to the inverted configuration of the ligands. In the Trp site, d-Trp forms no interaction with the main chain carbonyl group of Thr240 and less favourable interactions with Asn237 when compared to the l-Trp binding mode. Investigation of the *Mtu*DAH7PS_N175A_ variant further supports the hypothesis that the lack of key interactions in the binding mode of the aromatic d-amino acids are responsible for the absence of an allosteric response, which gives further insight into which residues of *Mtu*DAH7PS play a key role in the transduction of the allosteric signal.

## Introduction

Biological systems display inherent chirality. The majority of naturally occurring proteins and peptides are synthesized from exclusively l-amino acids, and only very limited significance of d-amino acids in biological systems was assumed. Recent studies have revealed that the utilization of d-amino acids in signaling and metabolism might be more widespread than previously thought [1, 2, 3, 4]. The discovery of d-Ser and its signaling function in mammalian brains [5], and the gene coding for the essential alanine racemase in *M*. *tuberculosis*, which converts l-Ala to d-Ala [6], are examples that reveal utilization of d-amino acids in animals. Although their handedness differs from l-peptides, peptides consisting of d-amino acids can display binding motifs that are recognized by biomolecules. However, d-peptides display increased stability in biological systems due to their increased resistance to proteolysis, a feature that has received some attention in inhibitor design [7, 8, 9].

The enzyme 3-deoxy-d-*arabino*-heptulosonate 7-phosphate synthase (DAH7PS) catalyzes the first committed step of the shikimate pathway, which is the aldol-like condensation of phosphoenolpyruvate (PEP) and erythrose 4-phosphate (E4P) to produce 3-deoxy-d-*arabino*-heptulosonate 7-phosphate (DAH7P). As the first enzyme, DAH7PS is a major control point for the shikimate pathway flux. Previous studies revealed a sophisticated synergistic allosteric regulation of DAH7PS from *Mycobacterium tuberculosis* (*Mtu*DAH7PS) by binary or ternary combinations of the three aromatic amino acids, Phe, Tyr and Trp. Binary combinations that involve Trp (Trp+Phe and Trp+Tyr) result in a significant loss of enzyme activity [10], and ternary combination of all three aromatic amino acids completely abolish the enzyme activity [11]. Adding an additional layer of sophisticated pathway flux regulation, *Mtu*DAH7PS forms a complex with chorismate mutase from the same organism [12, 13]. Chorismate mutase is an important branchpoint enzyme downstream of the shikimate pathway, catalyzing the transformation of chorismate to prephenate, the first committed precursor for phenylalanine and tyrosine biosynthesis. Formation and disruption of the *Mtu*DAH7PS-chorismate mutase complex allows the transfer of allosteric regulation to the otherwise unregulated chorismate mutase [14, 15]. This complex allosteric regulation has so far only been extensively studied for the DAH7PS enzyme from the pathogen *M*. *tuberculosis*. *Mtu*DAH7PS belongs to the type II DAH7PS group, in contrast to the far more common type I group that shows completely different allosteric inhibition. While from sequence homology it is expected that only the type II enzymes have the structural features to support this sophisticated allostery, it is clear not all type II DAH7PS enzymes are the same [16]. It might therefore be possible to exploit this system to provide new avenues for tuberculosis drug design.

**Fig 1:**
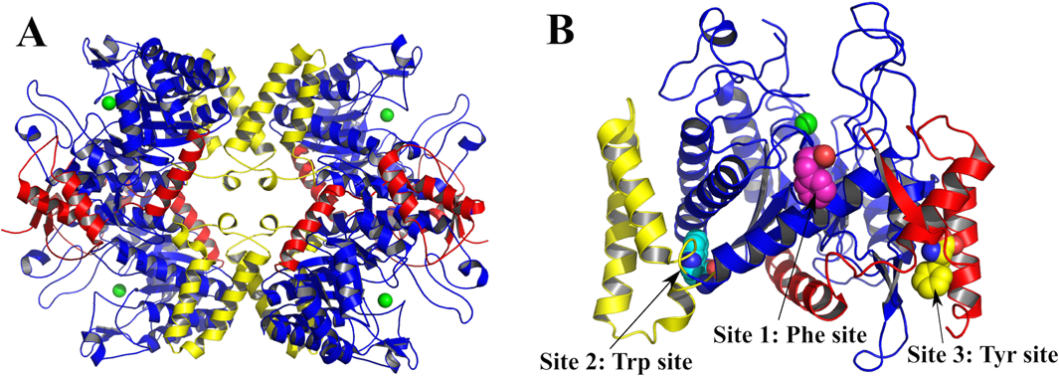
Structure of *M*. *tuberculosis* DAH7PS. (A) *Mtu*DAH7PS tetramer (PDB code 3NV8), the N-terminal extension (red) and α2β3- insertion (yellow) play key roles in the quaternary structure assembly. The core barrel residues are shown in blue, the active site metal ion is shown as a green sphere. (B) Allosteric binding sites of *Mtu*DAH7PS (PDB code 3KGF): in this structure, Phe occupies site 1 (purple spheres) and site 3 (yellow spheres). Trp occupies site 2 (cyan spheres).

The crystal structure of *Mtu*DAH7PS revealed that the quaternary structure of this enzyme is a homotetramer of (β/α)_8_ TIM barrel subunits (Fig 1A) [10]. Crystal structures of *Mtu*DAH7PS in complex with different combinations of aromatic amino acids revealed three distinctive allosteric binding sites located at the dimer and tetramer interfaces of the enzyme, formed by accessory elements to the basic barrel (Fig 1B) [10]. Site 1 is located near the dimer interface and has contributions from the N-terminal β0 strand. Site 2 is located near the tetramer interface and residues from the inserted α2a and α2b helices contribute to the binding site. Site 3 is nestled between the α0a and α0b helices of the N-terminal extension and the α3 helix of the core barrel. Previous studies combining crystallography and mutagenesis experiments at the allosteric binding sites established that while all the binding sites are promiscuous with respect to binding the three aromatic amino acids, site 1 is selective for Phe, site 2 is the Trp binding site, and site 3 is selective for Tyr, respectively [11].

To further probe this highly sophisticated allosteric regulation observed in *Mtu*DAH7PS, and to help inform drug design that targets the allosteric sites, the effect of aromatic d-amino acids on the activity and regulation of *Mtu*DAH7PS was investigated. Arguably, using the enantiomers of the native ligands is the most subtle modification possible in the discovery of structure-activity relationships of the allosteric binding sites of *Mtu*DAH7PS. Understanding the selectivity of the binding sites and how the allosteric signal is communicated between the multiple binding sites of this enzyme has implications for the design of alternative ligands as potential allosteric inhibitors.

## Results

### Aromatic d-amino acids do not elicit an inhibitory response

**Fig 2:**
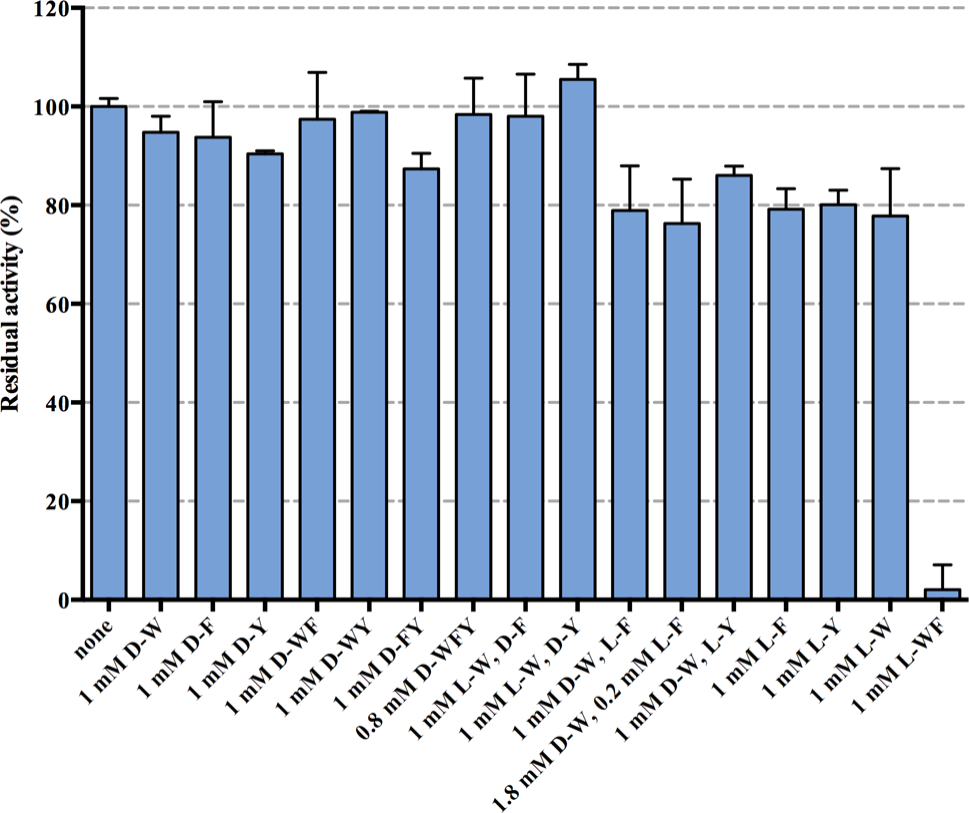
Effect of aromatic d-amino acids and combinations of d- and l-amino acids on *Mtu*DAH7PS activity. Error bars represent standard deviation of replicates, all activities are normalized to the activity obtained without addition of any aromatic amino acids. The triple combination of d-WFY was conducted at 0.8 mM concentration each to avoid non-linear effects at high absorbance in the photometric assay. The concentration of both PEP and E4P was 150 μM. W = tryptophan, F = phenylalanine, Y = tyrosine.

The response of *Mtu*DAH7PS to the presence of aromatic d-amino acids was examined. Initial rates of the enzymatic reaction were determined at a concentration of PEP and E4P of 150 μM in the absence and presence of various combinations of aromatic d-amino acids and combinations of d- and l- amino acids (Fig 2).

No combination of aromatic d-amino acids elicits a significant inhibitory response. Some reduction (78% residual activity) in activity was observed in the presence of d-Trp when l-Phe or l-Tyr are also present. However, the activities obtained in the presence of 1 mM d-Trp/1 mM l-Phe, 1 mM d-Trp/1 mM l-Tyr are very similar to the residual activities in the control experiments containing only 1 mM l-Phe or 1 mM l-Tyr respectively. This suggests that d-Trp has little effect even in the presence of l-Phe or l-Tyr, and certainly does not show synergistic inhibition as observed in previous studies with l-Trp/l-Phe or l-Tyr combinations [10, 11].

### Isothermal titration calorimetry confirms binding of aromatic d-amino acids

**Fig 3:**
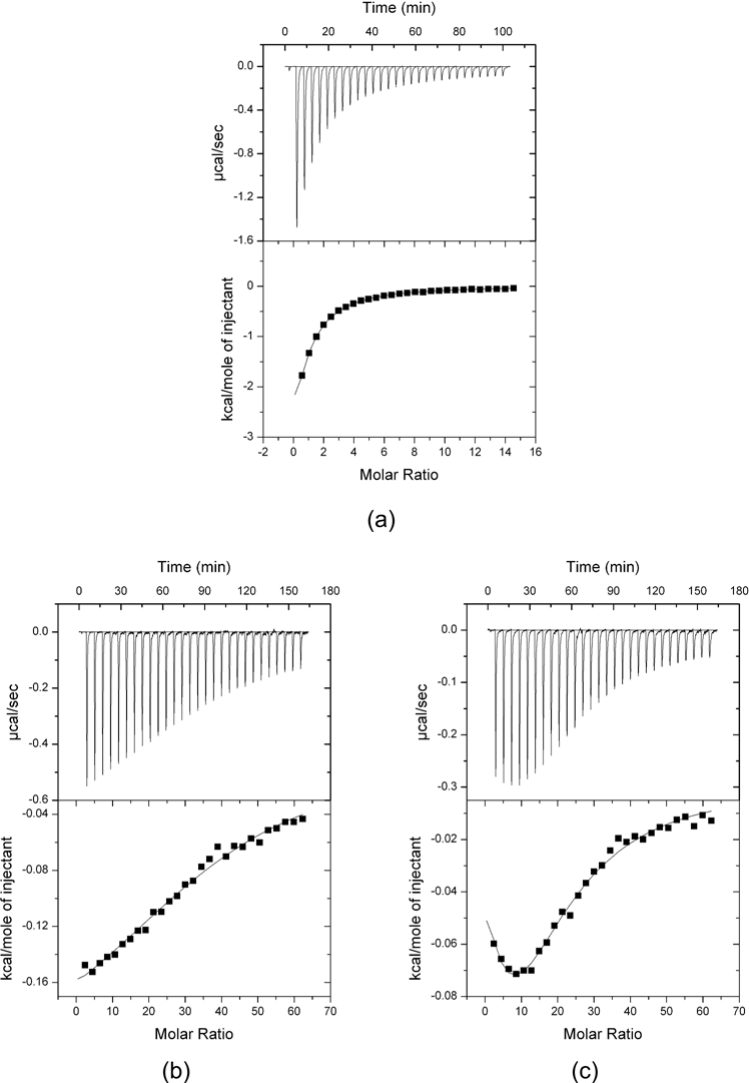
ITC data. Titrations of d-Phe (a) and d-Trp ((b), (c)) into a cell containing 5 μM *Mtu*DAH7PS tetramer, corrected for heats of dilution. (a) d-Phe (syringe concentration 3 mM) titration into a cell containing *Mtu*DAH7PS: fit of a one-site model to the data gives a *K*_d_ of 93 ± 5 μM. (b) d-Trp (syringe concentration 12 mM) titration into a cell containing *Mtu*DAH7PS: fit of a one-site model to the data gives a *K*_d_ of 700 ± 150 μM. (c) d-Trp (syringe concentration 12 mM) titration into a cell containing *Mtu*DAH7PS in the presence of 200 μM l-Phe: fit of a sequential two-site model to the data gives dissociation constants of 550 ± 60 μM and 1500 ± 330 μM.

In order to investigate whether the lack of inhibition of *Mtu*DAH7PS by the aromatic d-amino acids was due to a reduction of the binding affinities to the enzyme, the binding constants of d-Phe and d-Trp to *Mtu*DAH7PS were measured by isothermal titration calorimetry (ITC, Fig 3). Solutions of d-Phe and d-Trp were titrated into the calorimeter cell containing *Mtu*DAH7PS and a binding curve fitted to the measured heat changes. The affinity of d-Phe to the enzyme is only slightly lower when compared with the natural enantiomer: the dissociation constant (*K*_d_) of d-Phe from *Mtu*DAH7PS was determined as 93 ± 5 μM (Fig 3A). This represents a 4.5-fold reduction in affinity when compared to the dissociation constant (*K*_d_) of l-Phe from the enzyme, which was determined as 21 ± 1 μM [17]. This dissociation constant suggests that at the concentrations of d-Phe (1 mM) used in the enzymatic activity assay, virtually all binding sites for d-Phe on the protein are occupied.

*Mtu*DAH7PS interacts tightly with the natural l-enantiomer of Trp, the dissociation constant is 4.7 ± 0.1 μM [17]. In contrast, the affinity of *Mtu*DAH7PS for the unnatural enantiomer d-Trp is significantly reduced to a *K*_d_ of 700 ± 150 μM (Fig 3B). As it has been observed that the presence of l-Phe can increase the affinity of the enzyme to l-Trp [17], it was therefore tested whether l-Phe has any influence on the binding constant of d-Trp. A solution of d-Trp was titrated into a cell containing enzyme in the presence of 200 μM l-Phe (Fig 3C). The change in the shape of the ITC curve suggests that the presence of l-Phe does influence the binding of d-Trp. Both an independent two-site model and a sequential two-site model provide a good fit to the data. The independent two-site model assumes the binding occurs at two distinct sites whose thermodynamic parameters are independent from each other, while the sequential two-site model is based on the assumption that the two sites are coupled. In light of the knowledge that presence of l-Phe modulates l-Trp binding and vice versa, it is reasonable to assume that the two binding sites are not independent. Fitting of a sequential two-site model to the ITC data gives the two dissociation constants as 550 ± 60 μM and 1500 ± 330 μM, respectively.

### Binding mode of d-amino acids shown by crystallography

In order to elucidate the binding mode of the aromatic d-amino acids in more detail, crystal structures of *Mtu*DAH7PS in complex with the aromatic d-amino acids were determined using single crystal X-ray diffraction. To this end, single crystals of *Mtu*DAH7PS were grown in the absence of d-amino acids using the established conditions [18, 19]. The crystals were subsequently transferred to droplets containing all components of the crystallization droplet with the addition of the respective aromatic d-amino acid or combinations of aromatic amino acids. Crystals were left to soak before they were cryo-protected and flash-frozen for analysis.

Five crystal structures of *Mtu*DAH7PS in complex with different aromatic d-amino acids were obtained using different soaking conditions (Table 1). As was observed for the l-enantiomers, there is no major conformational change associated with binding of any of the d-amino acids to *Mtu*DAH7PS.

**Table 1:** Summary of soaking conditions and ligand occupancy for crystals soaked in d-amino acids.

Ligand(s)	pdb code	Soaking conditions	Site 1 ligand	Site 2 ligand	Site 3 ligand
**d-Phe**	5E2L	2.5 mM, 24 h	d-Phe	Water/ions	Water/ions
**d-Tyr**	5E40	1.6 mM final ^a^	d-Tyr	Water/ions	Water/ions
**d-Tyr**	5E4N	5 mM, 24 h	d-Tyr	Water/ions	d-Tyr
**l-Phe, d/l-Trp**	5E7Z	0.25 mM l-Phe, 2.5 mM d**/**l-Trp, 24 h ^b^	l-Phe	d/l-Trp	Water/ions
**d-Trp**	5E5G	10 mM d-Trp, 24 h	d-Trp	d-Trp	Waters/Ions

^a^ two additions of 0.2 μL of a 10 mM D-Tyr stock solution to a 2 μL drop, 24 hours between first and second addition and 24 hours between second addition and crystal freezing.

^b^ soaked in l-Phe and d-Trp (Acros, 92%ee by HPLC), which resulted in electron density in the Trp site which was modelled as d- and l-Trp at 50% occupancy each.

#### d-Trp and l-Phe can bind simultaneously to MtuDAH7PS

Early soaking experiments used l-Phe (0.25 mM) from Sigma Aldrich and d-Trp (2.5 mM) from Acros. After obtaining electron density in the Trp site, which suggested the presence of a significant amount of l-Trp, we carried out our own chiral HPLC analysis which revealed the d-Trp to be of only 92% ee. Considering the high soaking concentrations used, the relative affinities of l- and d-Trp determined by ITC and the electron density observed in the Trp site, we modelled the Trp site ligand as a mixture of l-Trp and d-Trp at 50% occupancy each. The Phe site in the structure obtained from these soaking conditions is occupied by l-Phe in a binding mode virtually identical to the l-Phe binding mode described in previous structures (data not shown). Further conclusions were precluded due to the ambiguous nature of the electron density of the Trp binding site and these data being superseded by soaking experiments with enantiomerically pure d-Trp. These preliminary findings however still suggested that d- and l-amino acids could simultaneously bind to *Mtu*DAH7PS and that lack of binding could not explain the lack of inhibition observed in kinetic assays.

#### Binding mode of aromatic d-amino acids in the Phe site

**Fig 4:**
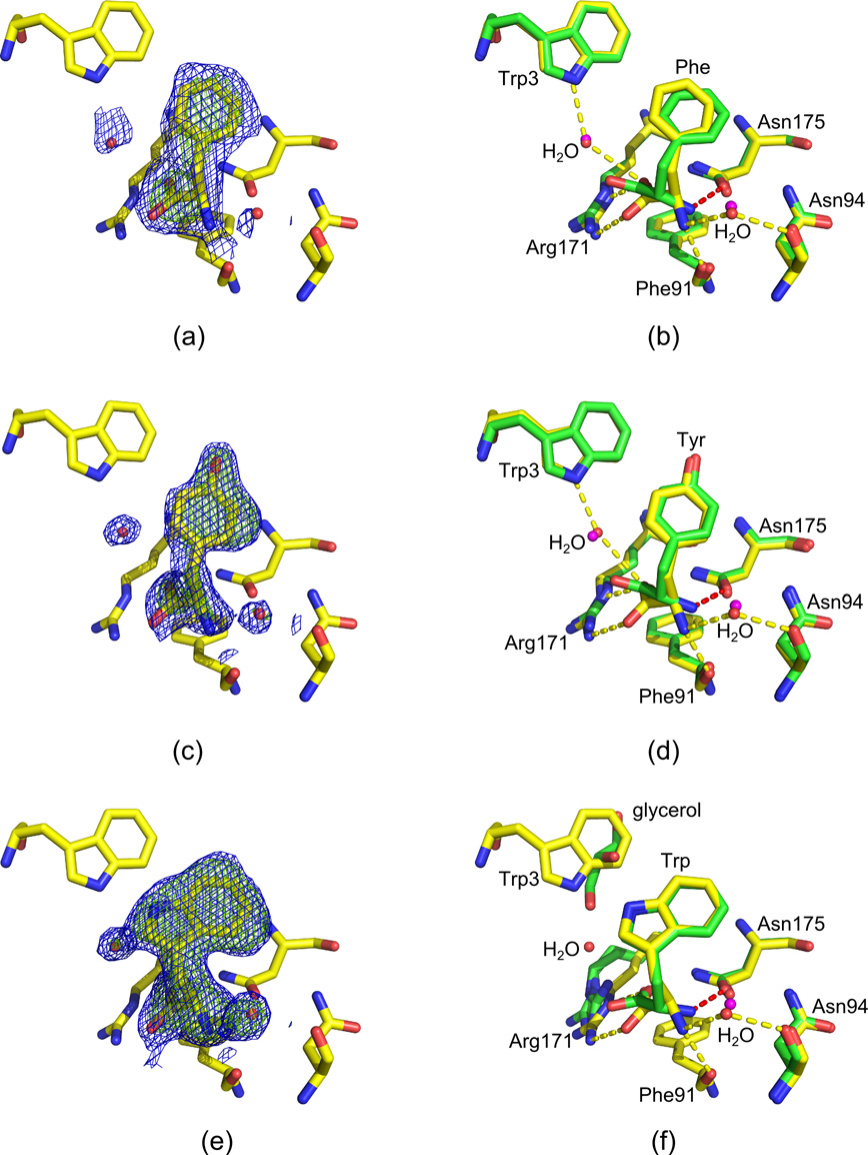
Binding modes of aromatic d-amino acids in the Phe site. Comparison of binding modes of d-Phe, d-Tyr and d-Trp (pdb id 5E2L, 5E4N and 5E5G respectively, shown as yellow sticks, waters shown as red spheres) with l-Phe, l-Tyr and l-Trp (pdb id 2YPO, 2YPP and 5EX4 respectively, shown as green sticks, waters shown as magenta spheres). The l-amino acids establish an additional hydrogen bond between the amino moiety of the ligand and the side chain carbonyl group of Asn175 on the protein (highlighted as red dashes) when compared to the d-amino acids (other interactions shown as yellow dashes). 2*F*_o_-*F*_c_ omit maps contoured at 1.0 σ (blue mesh) and *F*_o_-*F*_c_ omit maps contoured at 3.0 σ (green mesh). (a) Omit map showing the density into which d-Phe and two water molecules were modelled. (b) Comparison of l-Phe and d-Phe binding mode in the Phe site. (c) Omit map showing the density into which d-Tyr and two water molecules were modelled. (d) Comparison of l-Tyr and d-Tyr binding mode in the Phe site. (e) Omit map showing the density into which d-Trp and two water molecules were modelled. (f) Comparison of l-Trp and d-Trp binding mode in the Phe site.

The binding modes of all three aromatic d-amino acids in the Phe site are very similar to the binding modes of the respective l-enantiomers (Fig 4). The configuration of the ligand does not appear to significantly influence the conformation of the *Mtu*DAH7PS residues that form the Phe binding site. For all aromatic d-amino acids, the amino and carboxy moieties project into the same binding pockets as the respective moieties of the l-amino acids. d-Phe binds to the Phe site in a very similar binding mode as the l-enantiomer, the root mean square deviation (RMSD) between the position of d-Phe and l-Phe ligands is 1.46 Å. The phenyl side chain of d-Phe is slightly tilted in the hydrophobic pocket when compared to the binding mode of l-Phe. Similar to Phe molecules, there is little difference between the overall binding mode of d-Tyr and l-Tyr and d-Trp and l-Trp in the Phe site: The position of the aromatic rings as well as the Cβ-atoms of d-Trp and d-Tyr are virtually identical when compared with l-Trp and l-Tyr (RMSD of 0.07 Å and 0.02 Å when comparing all atoms of d-Tyr with all atoms of l-Tyr and d-Trp and l-Trp, respectively). Interestingly, while binding of l-Trp induces a conformational shift in the N-terminal region of the protein which is observed in all structures with l-amino acids and d-Phe and d-Tyr bound in site 1 [10], binding of d-Trp does not appear to induce a comparable rearrangement of the N-terminus. However, high atomic displacement factors and poor electron density for the N-terminal region in the d-Trp bound structure suggest that flexibility of this region might be increased upon ligand binding. The only major difference in binding mode when comparing aromatic amino acids of l-configuration with their respective d-enantiomers is the lack of a hydrogen bonding interaction between the amino moiety of the d-configured ligands and the sidechain carbonyl of Asn175 on *Mtu*DAH7PS (highlighted in red in Fig 4). The distance of the amino group of the ligand to the side-chain carbonyl group of Asn175 increases from 2.8 Å to 3.6 Å (l-Phe versus d-Phe), 2.6 Å to 3.7 Å (l-Trp versus d-Trp) and 2.7 Å to 3.6 Å (l-Tyr versus d-Tyr), respectively. In summary, while l-amino acids form a hydrogen bond between the amino moiety and the side chain of Asn175, the geometry of d-amino acids does not allow this interaction to be established (Fig 4B, 4D and 4F).

#### Binding mode of d-Trp in the Trp site

**Fig 5:**
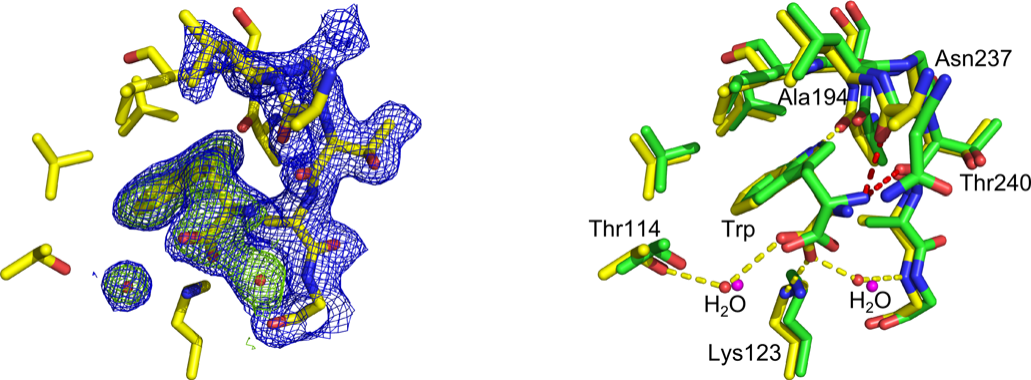
d-Trp in site 1. Left: Part of the electron density map in site 2 with d-Trp bound. The model conformation for the α2bβ3-loop is in good agreement with the map (2*F*_o_-*F*_c_ map contoured at 1.0 σ (blue mesh) and *F*_o_-*F*_c_ omit map contoured at 3.0 σ (green mesh)). Right: Comparison of binding mode of l-Trp (green sticks, water molecules shown as magenta spheres) and d-Trp (yellow sticks, water molecules shown as red spheres) in site 2. Key interactions of d-Trp are highlighted with yellow dashes. Red dashes indicate hydrogen bonds formed between the l-Trp amino group and Thr240 and Asn237. Due to the inversion of configuration, d-Trp does not establish a hydrogen bond to Thr240, and the hydrogen bonding interaction with Asn237 is of less optimal geometry compared to l-Trp.

Soaking of crystals of *Mtu*DAH7PS with 10 mM d-Trp yielded a structure with d-Trp bound in the Phe site and the Trp site (Table 1, pdb id 5E5G). Two key interactions observed in the binding mode of l-Trp in the Trp site are also found in the binding mode of d-Trp: the indole N-H of both enantiomers of Trp establishes a buried hydrogen bond to the Ala192 backbone carbonyl, and the carboxylate moieties of both enantiomers of Trp form salt bridges and hydrogen bonds to the side-chain ammonium group of Lys123. However, the inverted configuration of d-Trp results in the amino moiety of d-Trp adopting a position, which does not allow the establishment of a hydrogen bond to the backbone carbonyl groups of Thr240 on the α2bβ3-loop. Furthermore, the interaction of the amino group of d-Trp with the backbone carbonyl moiety of Asn237 is of less optimal hydrogen bonding geometry when compared to the interaction established by l-Trp (red dashes, Fig 5). Despite the less optimal geometry of the interactions of d-Trp with the α2bβ3-loop, the rearrangement of this loop typically observed upon l-Trp binding is also observed upon binding of d-Trp, and the electron density map and atomic displacement factors suggest a lowered flexibility of the loop upon ligand binding.

#### Binding mode of d-Tyr in site 3

**Fig 6:**
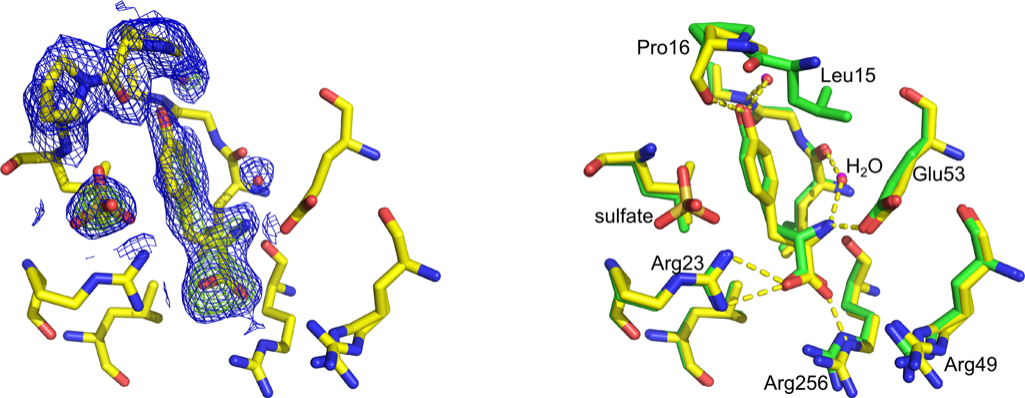
d-Tyr in the Tyr site (pdb id 5E4N). Left: Part of the electron density map in site 3 with d-Tyr bound. The model conformation for the Pro16 loop is in good agreement with the map, the omit map shows the density for d-Tyr, water molecules and a sulfate ion. 2Fo-Fc map contoured at 1.0 σ (blue mesh) and Fo-Fc omit map contoured at 3.0 σ (green mesh). Right: Comparison of l-Tyr (green sticks, water molecules shown as magenta spheres) and d-Tyr (yellow sticks, water molecules shown as red spheres) binding mode in the Tyr site. The sulfate ion is shown in gold (sulfur) and red (oxygen).

There is little difference in the overall binding mode of d-Tyr compared to l-Tyr in site 3 (Fig 6). This is reflected in the low RMSD of 0.16 Å when comparing all atoms of the d-Tyr ligand to all atoms of the l-Tyr ligand in site 3. One minor difference in the binding mode of d-Tyr is the distance between the phenolic oxygen of the d-Tyr side chain to the backbone carbonyl of Pro16, which increased from 2.6 Å found in the binding mode of l-Tyr to 3.1 Å in the d-Tyr-bound structure. However, the loop containing Pro16 shows disorder and high atomic displacement factors even in the presence of Tyr so that this discrepancy could also be due to this disorder and weak electron density for the residues concerned.

### Substitution of Asn175 with Ala disrupts allosteric regulation of *Mtu*DAH7PS by combinations of aromatic amino acids involving Phe

Crystallographic analysis revealed the difference in binding mode between the d- and l-amino acids in the three distinctive allosteric binding sites on *Mtu*DAH7PS. The d-amino acids are unable to establish hydrogen bond interactions with Asn175 in site 1, as well as Asn237 and Thr240 in site 2. This lack of interactions may be the cause for the lack of allosteric inhibition by d-amino acids. Since the only interaction involving side chain functionality is the hydrogen bond between Phe or Tyr and Asn175 in site 1, mutagenesis was employed to probe the role of Asn175 in the allosteric regulation of *Mtu*DAH7PS.

**Fig 7:**
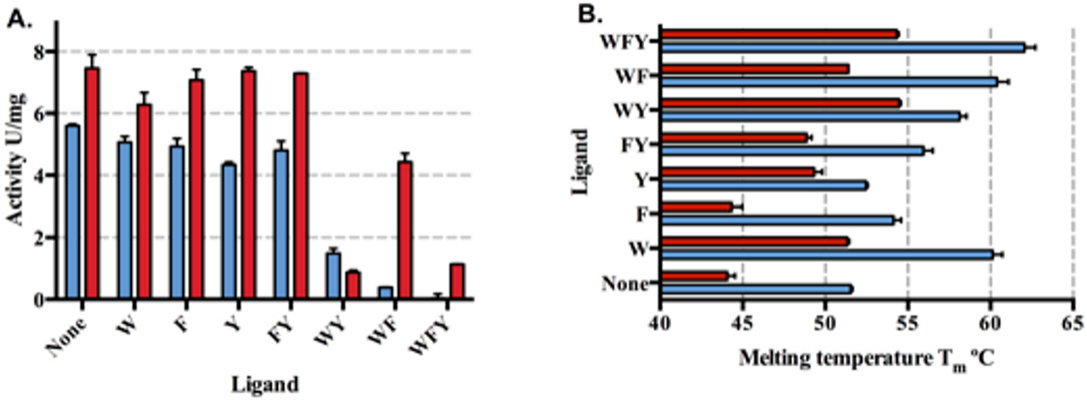
Inhibition of *Mtu*DAH7PS_N175A_ by aromatic amino acids compared to wild type enzyme. (A) Remaining activity of *Mtu*DAH7PS (blue) and *Mtu*DAH7PS_N175A_ (red) in the presence of various single, binary and ternary combinations of aromatic l-amino acids. Assays conducted in the presence of 150 μM E4P and PEP. Error bars depict the standard deviation of triplicate measurements. (B) The effect of single, binary and ternary combinations of aromatic l-amino acids on the thermal stability of *Mtu*DAH7PS (blue) and *Mtu*DAH7PS_N175A_ (red). The aromatic amino acids are represented by one letter code. Each letter also represents 200 μM of the corresponding aromatic l-amino acids.

Asn175 was substituted with Ala to remove its side chain functionality. Kinetic analysis of the resultant variant enzyme (*Mtu*DAH7PS_N175A_) showed that the increase in *k*_cat_ was accompanied by an increase in the *K*_m_ values for both E4P and PEP, so that the catalytic efficiency was not significantly affected (Table 2). Feedback inhibition studies showed that *Mtu*DAH7PS_N175A_ had significantly reduced sensitivity to l-Phe and slightly increased sensitivity to l-Trp and l-Tyr. Combinations of aromatic amino acids involving Phe showed less inhibitory effect to *Mtu*DAH7PS_N175A_ compared to the wild type (Fig 7A).

**Table 2:** Kinetic parameters determined from the Michaelis-Menten kinetics of *Mtu*DAH7PS_N175A_.

*Mtu*DAH7PS	*K*_m_^E4P^ (μM)	*K*_m_^PEP^ (μM)	*k*_cat_ (s^-1^)	*k*_cat_/ *K*_m_^E4P^ (s^-1^mM^-1^)	*k*_cat_/ *K*_m_^PEP^ (s^-1^mM^-1^)
**Wild type**	28 ± 2	37 ± 4	4.7 ± 0.1	170 ± 20	130 ± 20
**N175A**	61 ± 8	89 ± 7	7.6 ± 0.2	150 ± 20	100 ± 10

Differential scanning fluorimetry (DSF) was conducted to examine the binding of aromatic amino acids to *Mtu*DAH7PS_N175A_ (Fig 7B). Relative to wild-type *Mtu*DAH7PS the melting temperature (T_m_) of *Mtu*DAH7PS_N175A_ was increased in the presence of Trp and Tyr, suggesting these ligands still bind to this enzyme variant. Tyr caused a bigger shift of T_m_ in *Mtu*DAH7PS_N175A_ (~5°C) compared to the wild type enzyme (~1°C), which suggested that the substitution of Asn175 does not negatively impact l-Tyr binding. In contrast, the presence of l-Phe did not change the thermal stability of *Mtu*DAH7PS_N175A_. The lack of change in thermal stability suggests that at a concentration of 200 μM l-Phe may not bind to *Mtu*DAH7PS_N175A_.

## Discussion

Handedness or homochirality is a property of all known biological systems. The majority of amino acids occurring in nature are the l-enantiomers, which correspond to the (*S*)-configuration for all proteinogenic amino acids apart from cysteine. In addition, the majority of naturally occurring carbohydrates are chiral and present as single enantiomers in the biological molecules for which they provide the building blocks, for example the sugars contained in the DNA backbone. The mismatch of the less common enantiomers of amino acids with the biological systems of opposite handedness has been exploited in biological chemistry, for example in protease-stable poly-d-peptides which disrupt the MDM2/p53 interaction important in some cancers [7, 8, 9]. The study of the interaction of the d-enantiomers of the aromatic allosteric regulators of *Mtu*DAH7PS can help understand the complex allosteric regulation and the influence of the ligands on the protein conformational ensemble as well as provide information for the design of potent allosteric inhibitors.

The kinetic data show clearly that no combination of aromatic d-amino acids leads to a significant reduction in enzymatic activity up to 1 mM concentration, indicating that *Mtu*DAH7PS possesses an efficient mechanism of discriminating between aromatic amino acids of different configuration. The only combinations tested which lead to an appreciable reduction in enzymatic activity (d-Trp/l-Phe and d-Trp/l-Tyr) were only modest in their effect compared to the responses observed for the analogous l-Trp/l-Phe and l-Trp/l-Tyr combinations [10, 11]. In addition, control experiments indicate that the response obtained with d-Trp/l-Phe and d-Trp/l-Tyr respectively is comparable to the response from 1 mM l-Phe or l-Tyr alone. This response could be due to promiscuous binding of l-Phe/l-Tyr to multiple sites when they are present at high concentrations. Previously published work shows that l-Phe and l-Tyr exhibit similar behaviour in ITC experiments [11, 17]. The lack of distinction in the behaviour in kinetic assays of d-Phe or d-Tyr to each other, as well as the similarity of their crystallographically determined binding modes to each other and their l-enantiomers implies that studying d-Phe and d-Trp binding to the enzyme is sufficient to gain a better understanding of the inhibition of the enzyme. In addition, previous studies by us and others [9, 13, 17] indicate that *Mtu*DAH7PS is most significantly inhibited by a combination of l-Trp and l-Phe or l-Tyr occupying the Trp and Phe site of the enzyme, with additional inhibition when all three aromatic amino acids are present and the Tyr binding site is also occupied.

The isothermal titration calorimetry experiments reported in this study show that *Mtu*DAH7PS binds d-Phe with only slightly reduced affinity when compared to l-Phe, while the affinity of the enzyme for d-Trp is significantly lower than for l-Trp. Furthermore, the modulation of d-Trp binding in the presence of l-Phe suggests that d-Trp still preferentially interacts with site 2, the preferred site for l-Trp. It is interesting to note that although d-Trp is not inhibitory in combination with l-Phe, the presence of l-Phe markedly influences the binding affinity of d-Trp, showing although inter-allosteric site communication is not affected, a specific set of interactions is required by a ligand binding in the Trp site in order to cause an inhibitory signal to be transmitted. The lower affinity for d-Trp as compared to l-Trp is not sufficient to explain the lack of inhibition in the presence of 200 μM l-Phe/1.8 mM d-Trp. From the binding constants it can be estimated that with this combination a significant portion of the enzyme will have both l-Phe and d-Trp bound (l-Phe is at approximately 10×*K*_d_ and d-Trp at 3×*K*_d_), yet no significant reduction in enzyme activity is observed. X-ray crystallographic studies on enzyme-ligand complexes furthermore established that the respective d-amino acid can still bind to the sites which had been established as the preferred sites for the corresponding l-amino acid.

The combined insight from kinetic analyses, binding experiments and crystallographic studies indicates that binding of the aromatic d-amino acids does not result in the same signal being communicated between the allosteric sites and/or between the allosteric sites and active sites. The subtle differences in binding mode displayed by the d-amino acids could provide some insight into the pathways responsible for communication of allosteric site occupancy. The only significant difference between the binding mode of l-Phe and d-Phe in site 1 is the lack of a hydrogen bond between d-Phe and the Asn175 side-chain carbonyl. This may indicate that the ligand in site 1 needs to establish a contact to Asn175 in order for transmission of a signal from site 1 to either site 2 or the active site to occur. Mutagenesis experiments substituting Asn175 with Ala significantly reduced sensitivity of the enzyme to the aromatic amino acid Phe, which suggested that Asn175 is critical for signaling the binding of Phe in site 1. The binding mode of d-Trp compared to l-Trp in site 2 also reveals the lack of two hydrogen bonding-interactions to the amino moiety of the d-Trp ligand: the d-configuration leads to a geometry which does not allow interaction of d-Trp with the backbone carbonyl groups of Asn237 and Thr240 on the α2bβ3-loop. Interestingly, a partial conformational rearrangement of the α2bβ3-loop containing Thr240, which occurs upon l-Trp binding, is still observed in the presence of d-Trp, which indicates that establishment of a hydrogen bond between Thr240 and the ligand is not the major or only driving force for this conformational change to occur.

The results we present both here and in previously reported studies [5, 13, 17] indicate that sole ligand binding to the allosteric sites of *Mtu*DAH7PS is not sufficient to elicit an allosteric response, suggesting that specific interactions have to be established between the allosteric ligand and the enzyme. As some of these interactions involve the atoms of the peptide backbone of the enzyme rather than side chains, site-directed mutagenesis is not a viable option to examine the importance of these interactions. d-Trp acts as a minimally altered probe, in comparison to the native ligand, allowing us to elucidate the key interactions between the Trp-site ligand and the main chain carbonyls of Thr240 and Asn237. This conclusion would not have been possible using site-directed mutagenesis or if the binding mode of d-Trp would have been more significantly changed compared to the natural ligand l-Trp. In addition, the physico-chemical properties of the aromatic d-amino acids, apart from their absolute configuration, are identical to the native l-ligands. This allows us to eliminate potentially confounding variables as much as possible when drawing conclusions about the origin of the pronounced effect of the rather subtle changes in binding mode of opposite enantiomers.

Considering protein dynamics when discussing the allosteric regulation of *Mtu*DAH7PS is of paramount importance: the absence of hydrogen bonding-interactions between d-Trp and both Asn237 and Thr240 is expected to have some impact on the dynamic behavior of the α2bβ3-loop. This difference in dynamic behavior might in turn lead to disruption of the occupancy signal from site 2 to site 1 and/or the active site, ultimately resulting in no allosteric regulation by d-Trp.

In conclusion, the lack of inhibition of *Mtu*DAH7PS by d-amino acids yet again illustrates the sophistication of the network for allosteric regulation displayed by this enzyme [5, 7, 8, 13, 17, 18]. Despite binding to the appropriate sites, subtle differences in binding mode of the d-amino acids and their implications on protein dynamics appear to prevent a response to the less common enantiomers. From a ligand and inhibitor design point of view, the interactions necessary for inhibition and communication of site occupancy were clarified and will aid in future inhibitor design.

## Materials and Methods

### Kinetic assay

Enzymatic activity of *Mtu*DAH7PS was assayed based on the method reported by Schoner and Hermann including our previously reported adaptations [2, 5, 6, 17, 18, 20]: the consumption of PEP was followed by monitoring the absorbance at a wavelength of 232 nm (the extinction coefficient for PEP is ε(PEP, 280 nm, 25°C) is 2800 M^-1^ cm^-1^) using a Varian Cary 100 UV spectrophotometer. Kinetic assays were performed in 1 cm, 2 mm or 1 mm path-length quartz cuvettes, depending on the absorbance of the assay mixture used. Reaction mixtures were prepared by pipetting appropriate volumes of stock solutions into the cuvette to give the desired assay concentrations: typical assays contained PEP (150 μM), E4P (150 μM), manganese sulfate (100 μM), *Mtu*DAH7PS and a volume of assay buffer (50 mM 1,3-*bis*(*tris*(hydroxymethyl)methylamino)propane pH 7.5, 1 mM *tris*(2-carboxyethyl)phosphine) to make up the appropriate volume (1 mL for 1 cm path-length cuvettes, 300 μL for 1 mm and 2 mm path-length cuvettes). Inhibition assays were conducted with the addition of varying concentrations of the combinations of the aromatic amino acids as indicated in Fig 2 (*Mtu*DAH7PS wild type), or 200 μM l-Trp, 200 μM l-Tyr, and/or 200 μM l-Phe (*Mtu*DAH7PS_N175A_). Reactions were initiated either by addition of enzyme or addition of E4P. In both cases, the reaction mixture with all components but the initiating component was incubated at 30°C and the absorbance followed in the spectrophotometer. Reactions were initiated once the absorbance was constant. Initial rates were determined using the slope of a linear least square fit to the UV trace from 0.5 min after initiation to 1 min after initiation (1 cm path-length cuvettes) or from 15 s after initiation to 45 s after initiation (shorter path-length cuvettes), respectively. Rate measurements were repeated at least twice or until at least two measurements which agreed within 10% standard deviation of each other were recorded. The errors for kinetic measurements were estimated as the standard deviation of replicates.

### d-Amino acids

d-Phe and d-Tyr were from Sigma-Aldrich and ≥98% enantiomeric excess by g.l.c. according to the supplier. d-Trp was obtained from Accela and the enantiomeric excess was ≥99% by chiral HPLC according to the supplier. d-Trp used for soaking of the D/L-Trp/L-Phe-structure (PDB ID 5E7Z) was obtained from Acros and had 92%ee according to HPLC analysis.

### Isothermal titration calorimetry

ITC experiments were carried out on a VP-ITC microcalorimeter (Microcal, GE Healthcare) at 298 K. Before each experiment, the protein was buffer exchanged into the same buffer that was used for the preparation of the ligand solution (50 mM 1,3-*bis*(*tris*(hydroxymethyl)methylamino)propane pH 7.5, 1 mM *tris*(2-carboxyethyl)phosphine). The pH of each ligand solution was adjusted to the pH of the original buffer solution after complete dissolution of the ligand. For all experiments, the cell contained a solution of *Mtu*DAH7PS and the syringe contained the ligand solution. Experiments consisted of 29 injections, one 2 μL injection and 28 subsequent injections of 10 μL each, using a reference power of 10 μCal s^-1^. The protein concentration was measured by UV absorption at 280 nm and all solutions were filtered and degassed in a vacuum immediately before use. Before the titration, the cell and the syringe were washed with buffer several times, and in addition, the syringe was washed with the ligand solution. For data analysis, the initial data point was deleted to allow for diffusion of the ligand across the needle tip during the initial equilibration period. In an independent experiment, heats of dilution were measured and subtracted from the titration data before curve fitting of the data using Origin (version 7.0, OriginLab®) using the models supplied by MicroCal.

### Crystallization and crystal soaking

Purification of *Mtu*DAH7PS and crystallization were carried out as previously described [18, 19]. In brief, crystals were obtained using a hanging drop vapor diffusion setup with the reservoir solution containing 0.1 M Tris-HCl at pH 7.5, 1.5 M ammonium sulfate, 12% v/v glycerol. The crystallization droplet contained protein at a concentration between 3–5 mg/mL and mixed with an equal amount of reservoir solution (typical droplet sizes used were 2 μL total). Crystals were soaked in droplets of solutions containing d-amino acids at concentrations stated in Table 1 that had been pre-equilibrated to the same reservoir solution as the crystallization droplet. Crystals were harvested directly from the soaking solution and shock-frozen by plunging into liquid nitrogen.

### Data collection and structure refinement

Diffraction data were collected at the MX1 and MX2 beamline at the Australian Synchrotron, Victoria, Australia [21]. Automated preliminary data processing was carried out using AutoRickshaw [22, 23]. Data processing was carried out using XDS [24] and Scala or Aimless [25, 26]. Molecular replacement using the *Mtu*DAH7PS structure with PDB ID 3NV8 was performed by MOLREP to obtain initial phases [27]. The final model was obtained by multiple iterations of model rebuilding in COOT [28] and refinement using REFMAC5 [29]. Model validation tools in COOT and MolProbity [30] were used to detect and correct unlikely geometries in the model. In the refinement of ligand-bound structures, the model of the protein in the absence of any ligands was refined and rebuilt until the model was in close agreement with the electron density map. At this stage, waters and ligands other than the ligands of interest were added to the model apart for the regions of the structure where the electron density suggested the presence of the ligands of interest. Waters were added into appropriate electron density if the water would have at least one hydrogen bond to the protein. Waters in secondary and higher shells were only added if the position of the water molecule allowed the establishment of an uninterrupted chain of interactions to a protein atom. After the model and electron density was judged in good agreement in all regions apart from the ligand binding site(s) of interest, the electron density in the ligand binding site(s) was examined. If appropriate electron density was present, the ligand of interest, waters and potential other adventitiously bound molecules were added to the model and the final rounds of refinement carried out. 2*F*o−*F*c and *F*o−*F*c omit maps shown in figures were generated using the program FFT (CCP4 program suite) before addition of the ligands to the model. Figures for protein structure visualization were generated using Pymol [31]. Collection and refinement data are provided in Tables 3 and 4.

**Table 3:** Data collection and refinement statistics for structures reported in this publication (see also Table 4).

pdb id	5E2L	5E40	5E4N
A. *Data collection*			
Crystal system	Trigonal	Trigonal	Trigonal
Space group	*P*3_2_21	*P*3_2_21	*P*3_2_21
Unit cell parameters			
*a* (Å)	203.99	205.45	205.74
*b* (Å)	203.99	205.45	205.74
*c* (Å)	66.90	66.80	66.64
Resolution range (Å)^a^	49.00–2.50 (2.57–2.50)	19.85–2.05 (2.09–2.05)	47.37–2.05 (2.09–2.05)
Resolution where *I*/σ(*I*) = 2.0	3.00	2.48	2.61
CC_1/2_ outer shell	0.770	0.608	0.625
Measurements	620384	762663	1152805
Unique reflections	55365	101083	101243
Redundancy	11.2 (11.5)	7.5 (7.5)	11.4 (11.3)
Completeness (%)	100.0 (100.0)	99.9 (100.0)	100.0 (100.0)
*I*/σ(*I*)	4.0 (0.9)	5.4 (0.8)	4.7 (0.7)
*R*_merge_ (%)	19.0 (85.0)	14.3 (102.6)	16.5 (123.3)
Wilson *B*-value (Å^2^)	35.6	27.5	31.5
B. *Refinement*			
Resolution (Å)	49.00–2.50 (2.57–2.50)	19.85–2.05 (2.10–2.05)	47.37–2.05 (2.10–2.05)
*R*_cryst_ (%)	15.6^b^	19.4	19.9
*R*_free_ (%)	17.4^b^	20.7	22.9
Amino acids (chain length 464 residues)	462 + 458, 7196 atom sites	462 + 456, 7641 atom sites	459 + 454, 7606 atom sites
Water molecules	82	537	541
Other	2 D-Phe ligands, 2 Mn^2+^, 5 Cl^-^, 5 SO_4_^2-^, 2 glycerol	2 D-Tyr ligands, 2 Mn^2+^, 5 SO_4_^2-^, 1 glycerol	3 D-Tyr ligands, 2 Mn^2+^, 4 Cl^-^, 4 SO_4_^2-^
Mean *B* (Å^2^)			
Protein	36.2	27.4	31.8
Water	30.3	38.7	35.2
Other	36.1	39.2	35.3
r.m.s.d. from target values			
Bond lengths (Å)	0.007	0.006	0.011
Bond angles (°)	1.105	1.115	1.452
Dihedral angles (°)	5.684	5.242	5.597
Ramachandran			
Most favoured (%)	97.6	98.0	97.0
Allowed (%)	2.2	2.0	2.9
Disallowed (%)	0.2	0.0	0.1

^a^ according to CC_1/2_>0.5 cutoff;

^b^ intensity-based twin refinement was used.

**Table 4:** Data collection and refinement statistics for structures reported in this publication (see also Table 3).

pdb id	5E7Z	5E5G
A. *Data collection*		
Crystal system	Trigonal	Trigonal
Space group	*P*3_2_21	*P*3_2_21
Unit cell parameters		
*a* (Å)	207.97	205.00
*b* (Å)	207.97	205.00
*c* (Å)	67.03	66.54
Resolution range (Å)^a^	45.03–2.33 (2.38–2.33)	47.25–1.95(1.98–1.95)
Resolution where *I*/σ(*I*) = 2.0	2.93	2.01
CC_1/2_ outer shell	0.793	0.518
Measurements	876944	1300419
Unique reflections	71044	116470
Redundancy	12.3 (12.7)	11.2(8.5)
Completeness (%)	100.0 (100.0)	100 (99.7)
*I*/σ(*I*)	4.2 (0.6)	14.1(1.5)
*R*_merge_ (%)	17.0 (133.4)	14.3(139.2)
Wilson *B*-value (Å^2^)	42.9	23.5
B. *Refinement*		
Resolution (Å)	47.55–2.33 (2.40–2.33)	47.25–1.95(2.00–1.95)
*R*_cryst_ (%)	13.7^b^	16.9
*R*_free_ (%)	16.1^b^	18.4
Amino acids (chain length 464 residues)	459 + 462, 7337 atom sites	458 + 456, 7696 atom sites
Water molecules	131	648
Other	2 D/L-Trp and 2 L-Phe ligands, 2 Mn^2+^, 4 SO_4_^2-^, 1 glycerol	4 D-Trp ligands, 2 Mn^2+^, 4 SO_4_^2-^
Mean *B* (Å^2^)		
Protein	35.3	31.1
Water	32.3	38.4
Other	30.7	35.3
r.m.s.d. from target values		
Bond lengths (Å)	0.019	0.003
Bond angles (°)	2.006	0.715
Dihedral angles (°)	6.542	7.446
Ramachandran		
Most favoured (%)	98.0	98.1
Allowed (%)	1.6	1.9
Disallowed (%)	0.4	0.0

^a^ according to CC_1/2_>0.5 cutoff;

^b^ intensity-based twin refinement was used.

### Differential scanning fluorometry

Differential scanning fluorometry (DSF) was performed using a BioRad iCycler iQ5 Multicolour Real-Time PCR Detection System. Assays were prepared in a 96 well plate in size exclusion buffer (50 mM 1,3-*bis*(*tris*(hydroxymethyl)methylamino)propane pH 7.5, 1 mM *tris*(2-carboxyethyl)phosphine) with an enzyme concentration of 1 mg/mL and ligand added as required to give a final concentration of 200 μM for each ligand added. SYBROrange dye was also added to the protein sample mixture. The sealed plate was subjected to a thermal melt program from 20–95°C in 0.2°C increments over 4 h. Each sample was prepared in triplicate and compared to control containing dye, ligand solution and no enzyme. Melting temperatures were determined as the temperature at which the greatest increase in fluorescence was measured. The errors for the reported melting temperature measurements were estimated as the standard deviation of replicates.
